# Vitamin D3 Serum Levels in Periodontitis Patients: A Case–Control Study

**DOI:** 10.3390/medicina58050585

**Published:** 2022-04-24

**Authors:** Iwona Olszewska-Czyz, Elena Firkova

**Affiliations:** 1 Department of Periodontology, Prophylaxis and Oral Pathology, Medical Faculty, Jagiellonian University, 31155 Krakow, Poland; 2 Department of Periodontology and Oral Diseases, Faculty of Dental Medicine, Medical University–Plovdiv, 4002 Plovdiv, Bulgaria; Elena.Firkova@mu-plovdiv.bg

**Keywords:** periodontitis, vitamin D3 level, vitamin D3 deficiency

## Abstract

*Background*: Periodontitis is a multifactorial disease characterized by bacterial-dysbiosis-associated, host-mediated inflammation, which results in the loss of the tooth-supporting tissues. Vitamin D3 plays an important role in the tissue homeostasis and its deficiency might have a negative effect on the periodontitis progression and treatment outcomes. *Objectives*: The aim of the study was to evaluate the vitamin D3 serum levels among patients with periodontitis and healthy subjects. *Materials and Methods*: A total of 100 generally healthy adult participants (50 diagnosed with periodontitis, 50 with healthy periodontium) were enrolled in the study. The periodontal clinical parameters were measured, radiographs were performed and the 25-hydroxy vitamin D (25(OH)D) test was used to assess vitamin D3 levels. *Results*: Vitamin D3 levels were found to be statistically significantly lower among periodontitis patients (31.34; SD = 5.62) compared with healthy controls (39.64; SD = 8.77). Vitamin D3 deficiency was corresponding to the stage and grade of the disease as well as the clinical attachment and bone loss. *Conclusion*: Adequate monitoring of the vitamin D3 serum levels and supplementation could be of benefit in periodontitis treatment.

## 1. Introduction

### 1.1. Background

Periodontitis is a multifactorial disease, characterized by bacterial-dysbiosis-associated, host-mediated inflammation, which results in the loss of the tooth-supporting tissues. It is a major public health problem due to its high prevalence and the potential consequences: the attachment, alveolar bone and teeth loss, impaired chewing and aesthetics, as well as decreased quality of life. Many factors—systemic, environmental and genetic—can directly or indirectly influence the initiation and progression of periodontitis at multiple levels, and so the maintenance of the oral health can be very challenging [1,2].

Knowledge of the periodontal health and disease characteristics and understanding the inflammatory and immune mechanisms in the affected tissues are critical for implementation of relevant treatment strategies [1]. In this context, vitamins, as essential for normal functioning nutraceuticals, are important to be considered in the aspect of maintaining the balance within the periodontal complex. Among them, vitamin D3 is of particular significance. In the past decades, its role in health and disease has gradually gained an interest. Numerous studies have demonstrated its beneficial and regulatory effects on musculoskeletal health, hypertension and cardiovascular and metabolic diseases, such as diabetes mellitus [3,4,5]. Its biological functions also include anti-inflammatory and immune-modulating effects [6,7]. A recent study by Grant et al. [8] hypothesized that people at risk of respiratory tract infections, including influenza and/or COVID-19, could consider taking vitamin D3 to reduce the viral replication rates, concentration of proinflammatory cytokines and chronic disease comorbidities.

Public awareness for vitamin D3 deficiency and its effects on systemic health is increasing worldwide. The deficiency usually results from lack of sun exposure or inadequate nutrition, and it is higher in women and elderly people [9]. Vitamin D is mainly derived from skin and from dietary sources. More than 90% of systemic vitamin D originates from the skin and around 10% originates from food intake. 7-dehydrocholesterol is converted in the skin by ultraviolet light (UVB) to vitamin D3. The dietary sources of vitamin D are oily fish, red meat, liver, egg yolks and fortified foods, such as some fat spreads and breakfast cereals [3,7]. The most common symptoms of vitamin D3 deficiency are more frequent infections, tiredness or fatigue, hair loss, muscle pain, bone and/or lower back pain, depression or low mood and slower healing of wounds [3]. There is no international consensus on the definition of optimal levels of serum concentration, deficiency or sufficiency of vitamin D3. Different advisory bodies define different thresholds of vitamin D3 deficiency but most of them accept a serum concentration of 25–30 nmol/L as the threshold for deficiency and 50–75 nmol/L as being sufficient for all age groups [9]. It should be noted that population references for vitamin D3 ranges vary widely depending on the ethnic background, age and geographic location of the studied populations and the sampling season. The prevalence of a low serum 25(OH)D concentration (<50 nmol/L) is high, more than 50% during winter in many European countries. The presence of severe vitamin D3 deficiency (below 25/30 nmol/L) in specific risk groups including young children, adolescents, pregnant women, older people (especially the institutionalized) and non-Western immigrants is reported in [10]. 

### 1.2. Vitamin D3 and Its Association with Periodontal Health and Periodontitis

Evidence suggests that vitamin D3 could be relevant for periodontal health and periodontitis. It takes part in a nonspecific immune response to bacterial biofilm via stimulation of gingival fibroblasts, periodontal ligament cells, monocytes, macrophages and neutrophils to produce and release peptides such as β–defensin and cathelicidin. These have broad antimicrobial activity against Gram-positive and Gram-negative bacteria and play role in chemotaxis, cytokines production, vascular permeability, wound healing and neutralization of bacterial endotoxins [11,12]. Vitamin D3 also takes part in specific immune responses by suppressing the proliferation of T-lymphocytes, secretion of immunoglobulins, transformation of B-lymphocytes into plasma cells and decreasing the secretion of IL-1, IL-6, IL-8, IL-12 and TNF-α cytokines [13]. These cytokines are released during the initiation and progression of periodontal disease, stimulating osteoclast activity and the resorption of the alveolar bone. Thus, vitamin D3 may decrease alveolar bone resorption and increase bone density or negatively affect the course and progression of periodontitis [14,15]. 

The literature regarding the impact of serum vitamin D3 levels on periodontal status is ambiguous [16,17]. The data, supporting the association between vitamin D3 levels and periodontitis, are still “inconclusive” at the moment [18], or do not provide direct evidence of any causal association between D3 deficiency and eventual increased risk of periodontitis [19]. Thus, there is a need for randomized clinical trials evaluating and comparing the levels of vitamin D3 serum levels in patients with different stages and grades of periodontitis compared with periodontally healthy controls. 

Considering all above aspects, a hypothesis of differences in vitamin D3 serum levels between patients with periodontitis and healthy individuals has been raised. Additionally, a question of differences in vitamin D3 serum levels among patients with various prognosis, stage and grade of periodontitis has been addressed.

### 1.3. Classification of Periodontal and Peri-Implant Diseases and Conditions

The diagnosis of periodontitis is based on the clinical and radiologic examination in accordance with the 2017 World Workshop on the Classification of Periodontal and Peri-Implant Disease and Conditions [20]. This new classification defines three new clinical entities: periodontal health, reduced periodontium in health and gingival inflammation in a patient treated for periodontitis. It also recognizes three periodontal diagnoses: necrotizing periodontal diseases, periodontitis and periodontitis as a manifestation of systemic diseases. The other important feature of this classification is the introduction of the concepts of staging and grading. Staging (I–IV) intends to classify the severity and extent of a patient’s disease based on the measurable amount of destroyed tissue as a result of periodontitis and to assess the specific factors that may attribute to the complexity of long-term case management. The initial stage is determined using clinical attachment loss (CAL). One or more complexity factors may shift the stage to a higher level [20]. Grading aims to indicate the rate of periodontitis progression [20].

### 1.4. Study Objective

The aim of the study was to evaluate vitamin D3 serum levels among patients with periodontitis and healthy subjects. The main research objective was to analyze whether periodontitis may be associated with different vitamin D3 levels than the control group and to assess if severity, complexity, distribution and risk of progression of periodontitis may be linked to vitamin D3 level.

### 1.5. Trial Design

**Figure 1:**
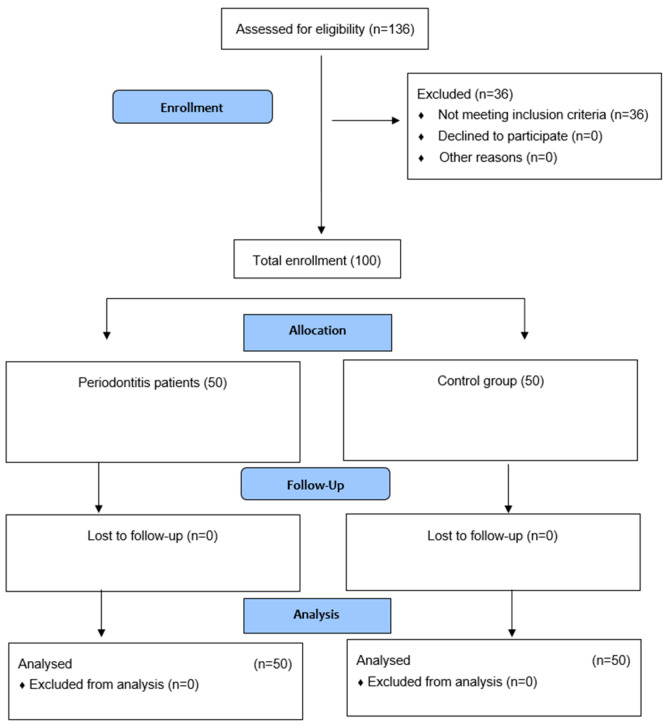
Consort flow diagram.

The trial was a single-center, study-control trial conducted at the Periodontology Department of University Dental Clinic in Cracow, Poland. The study was performed in accordance with the Helsinki Declaration. All the participants gave written informed consent to participate in the study. Official approval from the Jagiellonian University Ethics Committee was obtained (No.1072.6120.313.2018). The participants were enrolled during the periodontal appointments. The trial design is presented in the diagram (Figure 1). After the trial, patients were referred for follow-up periodontal care or treatment as needed.

## 2. Materials and Methods

### 2.1. Participants

A total of 100 generally healthy adult participants within normal BMI (50 diagnosed with periodontitis, 50 with healthy periodontium) were enrolled in the study. None of the participants took any antibiotics for the past 6 months or any nonsteroid anti-inflammatory drugs, corticosteroids or multivitamin supplements within the last 3 months. They had to be nonsmokers who were free from caries, epithelial dysplasia and inflammatory lesions of the oral mucosa. Pregnancy and receiving periodontal treatment, pharmacologic or radiologic therapy in the prior 6 months before the study were also added to the exclusion criteria. The diagnosis of periodontitis was based on the clinical and radiologic examination in accordance with the 2017 World Workshop on the Classification of Periodontal and Peri-Implant Disease and Conditions [20]. Staging (I–IV) intends to classify the severity and extent of a patient’s disease based on the measurable amount of destroyed tissue as a result of periodontitis and to assess the specific factors that may attribute to the complexity of long-term case management. Initial stage is determined using clinical attachment loss (CAL). One or more complexity factors may shift the stage to a higher level [20]. Grading aims to indicate the rate of periodontitis progression [20].

### 2.2. Data Collection

Data were collected at the baseline during scheduled periodontal appointment. Demographics, medical history, medication use, smoking habit and oral hygiene routine were recorded. The periodontal clinical parameters were measured, and radiographs were performed (Orthopantomagram by RAYSCAN Alpha P). A single periodontal examiner performed the following oral examinations: approximal plaque index (API) [21], bleeding on probing (BoP) [22], pocket probing depth (PPD), clinical attachment level (CAL). The instrument used was a periodontal probe (PCP-UNC 15, Hu-Friedy, Chicago, IL, USA). Missing teeth and alveolar bone level (ABL) were assessed (DICOM Viewer Software). ABL was assessed at the worst side in percentage according to the New Classification Guidelines. All clinical parameters were recorded in periodontal chart and analyzed according to stages and grades [20]. An indirect method of evidence to assess the grade/the progression was used: % of bone loss/age and case phenotype. 

Certified nurses collected 4 mL of venous blood from the participants at the baseline of the study after clinical data were collected. The patients received pre- and postintervention instructions. The 25-hydroxy vitamin D (25(OH)D) test was used to assess vitamin D3 level.

### 2.3. Statistical Methods

The data analysis was performed using the statistical software IBM SPSS version 27 (2020). In order to obtain the effect size of 0.5 with the level of significance set to 0.05 and the power set to 0.9, the calculated minimum required sample size was 47 subjects per group. Continuously measured variables were screened for normality through the Kolmogorov–Smirnov test and described through the means and standard deviations (±SD) when normality was observed. Student’s independent-samples *t*-test was used to compare the serum levels of vitamin D3 in the patients with periodontitis vs. the healthy controls and between target subgroups of the two main groups. When Levene’s test for equality of variances showed deviations (*p* < 0.05), the *p*-value for equal variances not assumed was reported. Within the periodontitis group, the effect of the stage, grade and distribution of the disease was examined through generalized linear model analyses, where the effect of each variable was adjusted for confounding influences by entering the remaining two as covariates. Associations of serum levels of vitamin D3 with other parameters were examined with Spearman rank–order correlation. The categorical variables were analyzed through the Chi-square test. All statistical tests were two-tailed and performed at level of significance alpha = 0.05. When multiple comparisons were carried out, the level of significance was adjusted through Bonferroni correction by the number of comparisons.

## 3. Results

### 3.1. Adverse Events and Safety Monitoring

All periodontitis patients enrolled in the study were followed by planned periodontal therapy after data collection. No adverse events were observed in any of the patients throughout the study.

### 3.2. Study Population

A total of 100 individuals (all Caucasian), 50 diagnosed with periodontitis and 50 evaluated as periodontally healthy, were enrolled in the study. The two groups had similar mean age and age range (*p* = 0.924) and almost equal distribution by sex (*p* = 0.841) (Table 1).

### 3.3. Serum Levels of Vitamin D3 in Patients with Periodontitis vs. Healthy Controls

The serum levels of vitamin D3 were consistently lower among the patients with periodontitis in the comparison with the healthy control group (31.34; SD ± 5.62 vs. 39.64; SD ± 8.77) regardless age and gender (Table 2).

### 3.4. Serum Levels of Vitamin D3 across Subgroups of the Patients with Periodontitis

**Figure 2:**
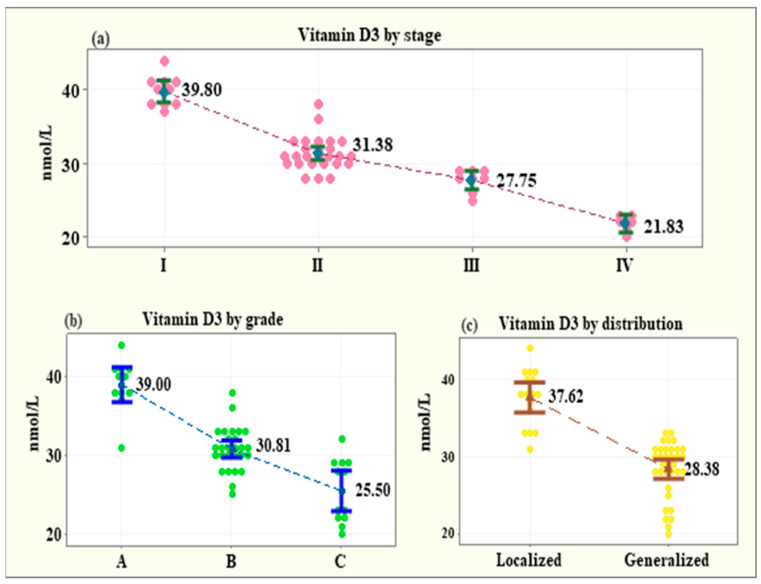
Vitamin D3 levels by the stage of periodontitis (**a**), the grade of periodontitis (**b**) and the distribution of periodontitis (**c**).

The stage of periodontitis (I–IV) was significantly negatively correlated with the serum level of vitamin D3. The vitamin D3 level decreased with the progression of the disease to a higher stage: Stage I (14 patients)—39.80 ± 2.09 nmol/L; Stage II (18 patients)—31.38 ± 2.24 nmol/L; Stage III (10 patients)—27.75 ± 1.48 nmol/L; Stage IV (8 patients)—21.83 ± 1.16 nmol/L (*p* < 0.001) (Figure 2a).

The grade (A, B and C) and the distribution of periodontitis (localized, generalized) were entered as covariates in a generalized linear model and a similar trend was observed. With grade and distribution as covariates, the mean vitamin D3 levels presented a significant reduction from Grade A to Grade C: Grade A (14 patients)—39.00 ± 3.31 nmol/L; Grade B (19 patients)—30.81 ± 2.76 nmol/L; Grade C (12 patients)—25.50 ± 4.03 nmol/L (*p* < 0.001) (Figure 2b). Additionally, the patients with generalized periodontitis had significantly lower mean level of vitamin D3 (28.38 ± 3.57 nmol/L, *p* < 0.001) in comparison with the patients with localized disease (37.63 ± 3.63 nmol/L, *p* < 0.001) (Figure 2c).

### 3.5. Associations between Vitamin D3 Levels and Clinical Parameters among Periodontitis Patients

**Figure 3:**
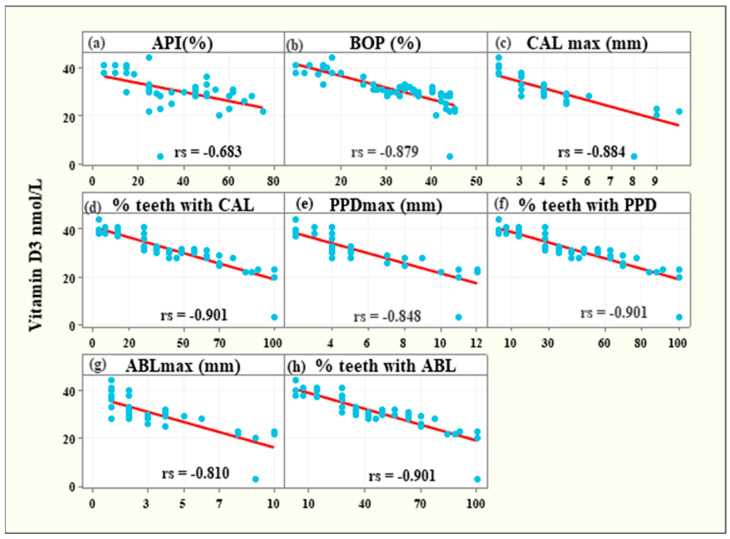
Strong negative associations between vitamin D3 levels and API (**a**), BOP (**b**), CALmax (**c**), % teeth with CAL (**d**), PPDmax (**e**), % teeth with PPD (**f**), ABLmax (**g**) and % teeth with ABL (**h**).

The serum levels of vitamin D3 were strongly negatively associated with the following parameters: APl (rs = −0.683, 95%CI: from −0.818 from to −0.475, *p* < 0.001), BOP (rs = −0.879, 95% CI: from −0.936 to −0.775, *p* < 0.001), the maximum CAL value (CAL max rs = −0.884, 95% CI: from −0.939 to −0.784, *p* < 0.001), the percentage of teeth with CAL due to periodontitis (% teeth with CAL rs = −0.901, 95% CI: from −0.948 to −0.814, *p* < 0.001), the maximum PPD value (PPD max rs = −0.848, 95% CI: from −0.919 to −0.724, *p* < 0.001), the percentage of teeth with PPD deeper than 3 mm (% teeth with PPD rs = −0.901, 95% CI: from −0.948 to −0.814, *p* < 0.001), the maximum ABL due to periodontitis (ABL max rs = −0.810, 95% CI: from −0.897 to −0.662, *p* < 0.001), the percentage of teeth with ABL (% teeth with ABL rs = −0.901, 95% CI: from −0.948 to −0.814, *p* < 0.001) (Figure 3).

## 4. Discussion

The results of current trial reported that the serum levels of vitamin D3 were statistically significantly lower among the patients with periodontitis in comparison with the healthy individuals (31.34 nmol/L; SD = 5.62 vs. 39.64 nmol/L; SD = 8.77) regardless age and gender. The association between serum levels of vitamin D3 and periodontal status has been investigated in many studies and lower levels of vitamin D3 are reported to be associated with periodontitis [22,23,24]. In a cross-sectional investigation by Bonnet et al. [23], vitamin D status was determined by 25(OH)D concentrations and a statistically significant association was observed between CAL and 25(OH)D levels lower than 75 nmol/L; however, the authors did not find such correlation with vitamin D levels lower than 50 nmol/L. The study by Madi et al. [24] aimed to assess the relationship between vitamin D levels and periodontal health among individuals in the Eastern Province of Saudi Arabia. A total of 67 participants were categorized according to vitamin D level (<10, <20 and >20 ng/mL) and their bone loss was compared. A negative correlation between vitamin D serum levels and ACH (alveolar crest height) was reported in all groups. In a meta-analysis, Machado et al. [25] compared vitamin D levels among periodontitis and periodontally healthy patients and assessed the influence of supplementation as an adjunctive to nonsurgical therapy. They took sixteen studies into account including fourteen case–control studies and two intervention trials. They concluded 25(OH)D levels were significantly lower in chronic periodontitis patients. In a trial by Antonoglou et al. [22], the correlation between 25(OH)D and 1,25(OH)_2_D serum levels and periodontal health was investigated. A total of 55 individuals with chronic periodontitis and 30 healthy patients were tested and a statistically significant association between 1,25(OH)_2_D serum level and periodontal health status was found. The correlation between periodontitis and 25(OH)D level was not confirmed; however, the study sample was a limitation of this finding. Due to the different methodology of previous mentioned studies—either the study samples, the inclusion criteria were different or the clinical parameters assessed were limited—it is difficult to proceed with a direct comparison to the current trial results; however, most of them reported a similar significant correlation between periodontitis and lower vitamin D levels.

In our study, the serum levels of vitamin D3 were strongly negatively associated with bleeding on probing, clinical attachment level, probing depth, alveolar bone loss and the percentage of teeth affected by bleeding on probing, probing depth deeper than 3 mm, attachment or bone loss. There are not many clinical trials evaluating so many parameters of periodontitis in the aspect of vitamin D level assessment. Balci Yuce et al. [26] evaluated proinflammatory cytokines and vitamin D levels in rheumatoid arthritis and chronic periodontitis patients and compared the status before and after initial periodontal treatment with healthy individuals. Serum and gingival crevicular fluid (GCF) vitamin levels were measured, and the authors concluded that vitamin D levels might be an important indicator of periodontal bone loss. In another study, it was concluded that low 25(OH)D concentrations are associated with periodontal disease; however, that was determined only by the gingival index (GI) and CAL measurements [23]. Lee et al. [27] investigated the relationship between vitamin D deficiency and periodontitis in Korean adults. Periodontal status was assessed by the community periodontal index (CPI) and showed no significant correlation with periodontitis based on CPI scores; however, this index has some limitations, as the extent of the inflammation is not addressed, and it lacks precise periodontal status evaluation. In a controlled study by Isola et al. [28], the impact of periodontitis and systemic sclerosis on vitamin D levels was investigated. Vitamin D level was negatively associated with probing depth, clinical attachment level and bleeding on probing. Patients with periodontitis had significantly lower vitamin D values than healthy individuals. Kim et al. [29] evaluated whether serum 25(OH)D levels are associated with periodontal disease status and tooth loss. The probing depth, clinical attachment level and bleeding on probing, as well as the percentages of sites with PD ≥ 4 mm and CAL ≥ 4 mm, were recorded in this study. The authors concluded that low serum 25(OH)D is significantly correlated to tooth loss and severe periodontitis.

The current results report that the stage of periodontitis (I–IV) was significantly negatively correlated with the serum level of vitamin D3, which decreased with the progression of the disease to a higher stage (Stage I—39.80 ± 2.09 nmol/L, Stage II—31.38 ± 2.24 nmol/L, Stage III—27.75 ± 1.48 nmol/L, Stage IV—21.83 ± 1.16 nmol/L). Similar findings refer to the grade (A, B and C) and the distribution of the disease (localized, generalized). The mean vitamin D3 levels presented a significant reduction from Grade A to Grade C (Grade A—39.00 ± 3.31 nmol/L; Grade B—30.81 ± 2.76 nmol/L; Grade C—25.50 ± 4.03 nmol/L) and individuals with generalized periodontitis had significantly lower mean level of vitamin D3 (28.38 ± 3.57 nmol/L) than patients with localized disease (37.63 ± 3.63 nmol/L). Most of the existing studies in this topic base their assessment methods on a previous periodontitis classification; thus, a direct comparison of the results might be inaccurate. However, most of the findings present the same trend of correlation between vitamin D levels and severity of the disease. In a case–control study by Alzahrani et al. [30], the association between periodontitis and vitamin D status among adults was examined. Sixty patients with severe or moderate periodontitis were included in the study and compared to a control group of sixty-three healthy individuals. The results reported significant correlation between periodontitis and serum levels of vitamin 25(OH)D. Costantini et al. [31] performed a comparison of inflammatory mediators and vitamin D levels in saliva of periodontitis individuals vs. healthy controls and reported highly negative correlation between 25(OH)D and periodontitis. Another trial [32] aimed to evaluate the relationship between 25(OH)D deficiency and severity of chronic periodontitis in type 2 diabetes individuals and found strong association between them. Moreover, the authors concluded that vitamin D deficiency may be related to the immune background of the disease. Agrawal et al. investigated the level of vitamin D of periodontally healthy chronic gingivitis and chronic periodontitis patients, with and without type 2 diabetes, and concluded that vitamin D level was contributing to increase in periodontal disease severity. A Norwegian study [33] also showed a significant association with periodontitis expressed by radiographic bone loss and vitamin D level. Our trial provides further evidence that vitamin D3 levels were significantly lower in the patients with periodontitis vs. the healthy control group. There was a correlation between the age of the patient, the stage and grade of periodontitis and the vitamin D3 level in this group. Patients older than 50 years with advanced (stage IV) and generalized periodontitis tend to have the lowest vitamin D3 levels. As periodontitis is a multifactorial disease, identification of risk factors is of utmost importance. Monitoring the serum levels of vitamin D3 in patients with periodontitis, especially older people with stage III and IV generalized periodontitis, and its adequate supplementation could potentially benefit the treatment outcomes and long-term prognosis. Further studies are necessary to assess the influence and dosage of vitamin D3 supplementation as an adjunctive to periodontal therapy on treatment outcomes.

The main strength of this controlled clinical trial was the evaluation of the vitamin D3 serum levels’ correlation with the severity, distribution, risk of progression and most important clinical parameters of periodontitis. To our knowledge, no such detailed analysis has been performed in previous studies. Another advantage of the trial is the participants selection criteria, as only generally healthy individuals were included in the study. The main limitation of the study is the sample size, which could have been higher, especially in terms of analyzing the differences between different stages of the disease and clinical parameters. Future investigations should definitely include more participants. Another limitation of the study is the single-time vitamin D3 level measurement, which does not permit long-term analysis. Although the authors put all efforts to keep the risk of bias on the minimum level, the limitation to a single clinical examiner might be a disadvantage of the study.

## 5. Conclusions

The current study reported reduced levels of vitamin D3 in periodontitis patients in comparison with healthy individuals. Moreover, within the limits of the study, it seems that the vitamin D3 serum level decreased with the higher severity, distribution and risk of progression of the disease. Further studies should take into account some other factors, especially the fact of the vitamin D3 serum levels’ changes with time rather than the limited single-time analysis.

## Figures and Tables

**Table 1:** Demographic information of the study population.

Variable	Periodontitis	Healthy Controls	*p*-Value
	*N* = 50	*N* = 50	
Age			
Mean ± SD	49.08 ± 10.14	49.28 ± 10.78	0.924 ^*t*^
Minimum–Maximum	25–65	29–62	
Sex *n* (%)			
	25 (50%)	24 (48%)	0.841 ^χ2^
Male	25 (50%)	26 (52%)	
Female			
BMI (Body Mass Index)			
Mean ± SD	22.07 ± 3.74	21.26 ± 4.18	0.724 ^*t*^
Minimum–Maximum	18.65–24.35	19.00–24.25	
BOP (Bleeding on Probing)			
Mean ± SD	31% ± 11%	31% ± 10%	0.001 ^*t*^
Minimum–Maximum	15–45%	12–47%	
PPD (Periodontal Pocket Depth)			
Mean ± SD	5.52 ± 2.64	2 ± 1	0.001 ^*t*^
Minimum–Maximum	4–12	0–2	
CAL (Clinical Attachment Level)			
Mean ± SD	4.14 ± 2.15	0	0.001 ^*t*^
Minimum–Maximum	2–10	0	

^*t*^—independent samples *t*-test; ^χ2^—Chi-square test.

**Table 2:** Mean serum levels of vitamin D3 (nmol/L) among patients with periodontitis vs. healthy controls.

Groups	*N*	Mean	Mean Diff.	*p*-Value
		(SD)	(95% CI)	
Overall				
o Periodontitis	50	31.34 (5.62)	8.3	<0.001 !
o Healthy controls	50	39.64 (8.77)	(5.37 to 11.22)	
Male				
o Periodontitis	25	31.64 (5.67)	8.23	0.001 !
o Healthy controls	24	39.88 (9.69)	(3.61 to 12.85)	
Female				
o Periodontitis	25	31.04 (5.67)	8.38	
o Healthy controls	26	39.42 (7.99)	(4.46 to 12.29)	<0.001 !
Age < 50 years				
o Periodontitis	22	32.50 (5.58)	7.63	0.002 !
o Healthy controls	22	40.14 (9.39)	(2.93 to 12.33)	
Age > 50 years				
o Periodontitis	28	30.43 (5.58)	8.82	<0.001 !
o Healthy controls	28	39.25 (8.38)	(5.00 to 12.63)	

Vitamin D3 levels were measured in nmol/L; !—*p*-values for equal variance not assumed in multiple independent-samples *t*-tests with Bonferroni adjusted level of significance *alpha* = 0.01.

## Data Availability

The data presented in this study are available on request from the corresponding author. The data are not publicly available due to ethical restrictions.
